# Role of Seagrass as a Food Source for Benthos in Tidal Flats: Toward Conservation and Restoration of Resilient Ecosystems

**DOI:** 10.3390/ani15081098

**Published:** 2025-04-10

**Authors:** Yumi Nagahama, Munehiro Nomura, Osamu Nishimura

**Affiliations:** 1 Ibaraki Kasumigaura Environmental Science Center, Tsuchiura 300-0023, Japan; 2 Department of Civil and Environmental Engineering, Graduate School of Engineering, Tohoku University, Sendai 980-8579, Japan; munehiro.nomura.d3@tohoku.ac.jp (M.N.); osamu.nishimura.d2@tohoku.ac.jp (O.N.)

**Keywords:** carbon and nitrogen stable isotope, fatty acid biomarkers, macrobenthos, particle organic matter, soil organic matter

## Abstract

Seagrass is a key primary producer in coastal ecosystems; however, most studies on seagrass-benthos interactions have focused on subtidal zones. Some species such as *Zostera japonica* grow in intertidal flats; however, their ecological functions remain unclear. Understanding whether intertidal seagrass beds contribute to benthic abundance and diversity can provide insights that facilitate tidal flat conservation. The present study clarifies the role of intertidal *Z. japonica* as a food source for benthos. Field surveys were conducted in an intertidal flat in Matsushima Bay, Japan. Five benthic species (*Batillaria cumingii*, *Umbonium costatum*, *Phacosoma japonicum*, Nereididae, and Paguroidea) were identified. Carbon and nitrogen stable isotope ratios (δ^13^C, δ^15^N) and fatty acid compositions of sediment organic matter, seawater, and target benthos were analyzed to determine food sources. The results showed that *B. cumingii* actively consumed *Z. japonica*-derived organic matter present in both seagrass and sandy sediments. *Z. japonica* also influenced bacterial community structure, providing a favorable habitat for Nereididae. Filter feeders (*U. costatum* and *P. japonicum*) exhibited minimal reliance on *Z. japonica*-derived organic matter. The findings suggest that, similar to subtidal seagrass ecosystems, intertidal seagrass meadows support benthic communities by supplying organic matter and enhancing bacterial production.

## Simple Summary

Seagrass plays an important role in coastal ecosystems by providing food and habitats for many marine animals. While most research has focused on seagrass growing in the subtidal zone, some species, such as *Zostera japonica,* grow in tidal flats that are exposed at low tide. However, their roles in supporting marine life are not well understood. This study investigated whether intertidal seagrass beds provide food for small animals inhabiting tidal flats. Field surveys were conducted in Matsushima Bay, Japan, where sediment, seawater, and five benthic species, including a mud snail (*Batillaria cumingii*), two types of bivalve (*Umbonium costatum* and *Phacosoma japonicum*), a marine worm (Nereididae), and small hermit crabs (Paguroidea) were collected. Dietary analyses revealed that mud snails and marine worms relied on organic matter from *Z. japonica*, whereas bivalves did not. Seagrass also influences bacterial communities in the sediment, establishing a favorable environment for marine worms. The results suggest that intertidal seagrass beds support marine life by supplying food and improving habitat conditions, similar to seagrass beds in the subtidal zone. Understanding the role of seagrasses could guide conservation and restoration efforts in tidal flat ecosystems.

## 1. Introduction

Tidal flats are widely distributed in Asia, North America, and South America, covering approximately 127,921 km^2^. Between 1984 and 2016, approximately 16% of these areas were lost due to coastal development, reduced sediment supply, and sea level rise [1]. Approximately 31% of the tidal flats were located within protected areas. However, tidal flats in Asia continue to decline [2]. In Wu Bay, a loss of 130 km^2^ was predicted between 1987 and 2021 [3]. Similarly, more than 40% of the tidal flats along the Japanese coast have been lost by land reclamation since 1945 [4]. However, as the importance of tidal flat ecosystem services has become clear, efforts toward conservation, restoration, and regeneration have been increasingly emphasized in recent years [4]. To continue benefiting from the ecosystem services, including supply services such as biomass production, and regulating services through enhanced ecosystem stability, it is essential to conserve and restore tidal flats. Biodiversity plays a key role in the processes since the coexistence of diverse species enhances ecosystem stability [5,6,7].

Seagrass is a major primary producer in coastal areas and has a significant impact on coastal ecosystems, owing to its high productivity [8]. Benthos is more abundant in seagrass beds than in adjacent non-seagrass areas [9,10,11,12]. One potential reason for this is the higher food supply capacity of seagrass meadows. Lee et al. [11] suggested that the canopy structure of seagrass enhances food availability through physical functions, such as detritus trapping, thereby increasing the abundance and diversity of the epibenthic community. In addition, according to Kharlamenko et al. [13], invertebrate communities within seagrass beds utilize organic carbon (C) derived from seagrass, *Zostera marina*, and benthic microalgae. Furthermore, they suggested that filter-feeding bivalves assimilate a considerable number of bacteria from resuspended sediments. Similarly, Alfaro [10] proposed that bacteria within seagrass beds are closely related to the organic matter in surface sediments and serve as an important food source for primary consumers.

However, these findings were based on seagrass beds in the subtidal zone. Some seagrass species, *Zostera noltii* or *Zostera japonica*, possess mechanisms of withstanding desiccation and grow in intertidal tidal flats [8]. The physical conditions of intertidal and subtidal zones differ significantly, leading to distinct biological communities and ecosystem structures. Seagrass canopies create habitats for benthic animals in intertidal zones exposed to rapid environmental changes. However, specific mechanisms underlying these processes remain unclear [14].

*Z. japonica* is widely distributed in Asia and occurs on tidal flats along the Japanese coast. As its productivity per unit area is as high as that of *Z. marina* [15], it may have a significant impact on material cycling in tidal flats, similar to that of subtidal seagrasses. However, seagrass beds formed in the intertidal flats do not necessarily play the same role as those in the subtidal zone. If seagrass beds in the intertidal zone also enhance the abundance and diversity of benthic organisms by supplying food resources, it could have key implications for the conservation and restoration of tidal flats.

This study aimed to clarify the role of intertidal seagrass as a food source for the benthos inhabiting tidal flats. Because many benthos are filter and deposit feeders, we focused on particulate organic matter (POM) in seawater and sedimentary organic matter (SOM) as potential food sources. To determine the origin and composition of organic matter, we analyzed the C and nitrogen (N) stable isotope ratios (SIRs), as well as fatty acid composition (FAC), and investigated the following aspects. (1) First, we examined the influence of *Z. japonica* on POM and SOM by comparing seagrass beds with nearby sandy areas where *Z. japonica* was absent. (2) Next, we identified representative benthos and assessed the effects of seagrass as a food source. By integrating the findings, we clarify the role of *Z. japonica* in tidal flat ecosystems.

## 2. Materials and Methods

### 2.1. Sample Collection

**Figure 1:**
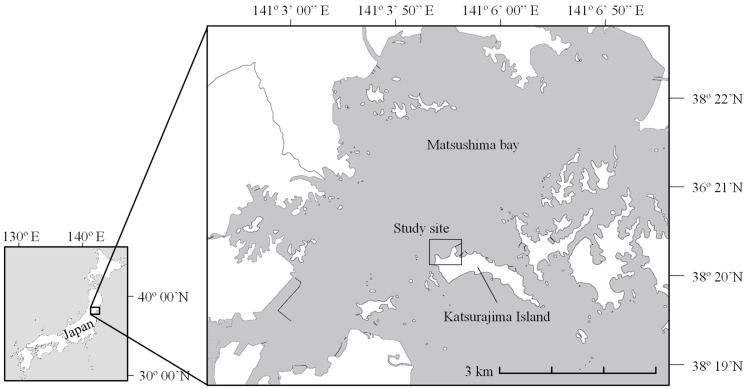
Schematic diagram of the study site in Matsushima bay, Katsurajima Island, Japan.

**Figure 2:**
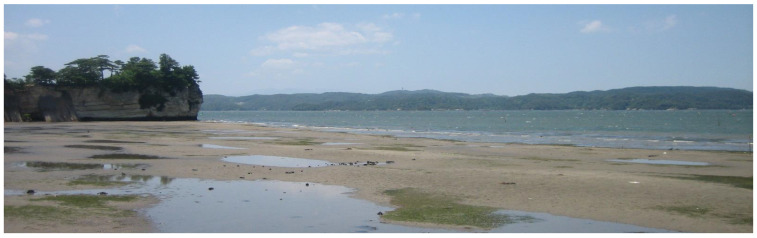
Photo of the study site with seagrass points and sand points.

The study site (38°20′7.4 N and 141°5′14.7 E) was located in the northwest of Katsurajima Island, in the central part of Matsushima Bay, Japan (Figure 1). Approximately 0.25 km^2^ of intertidal flats were covered by patchy seagrass beds of *Z. japonica,* each several meters in diameter. Based on previous studies conducted at the same site, the shoot density of *Z. japonica* ranged from 20 to 11,130 shoots/m^2^, with an annual mean of approximately 2920 shoots/m^2^. Although the aboveground biomass of *Z. japonica* showed significant seasonal variation, its belowground biomass remained relatively stable throughout the year [12]. In the subtidal zone, *Z. marina* grew continuously and formed a rectangular belt several meters wide and tens of meters long along the shoreline. There are no river inflows to the site, and the area is backed by steep cliffs. The sediment was sandy, with a median grain size (D_50_) of 0.018 mm [12] (Figure 2).

**Figure 3:**
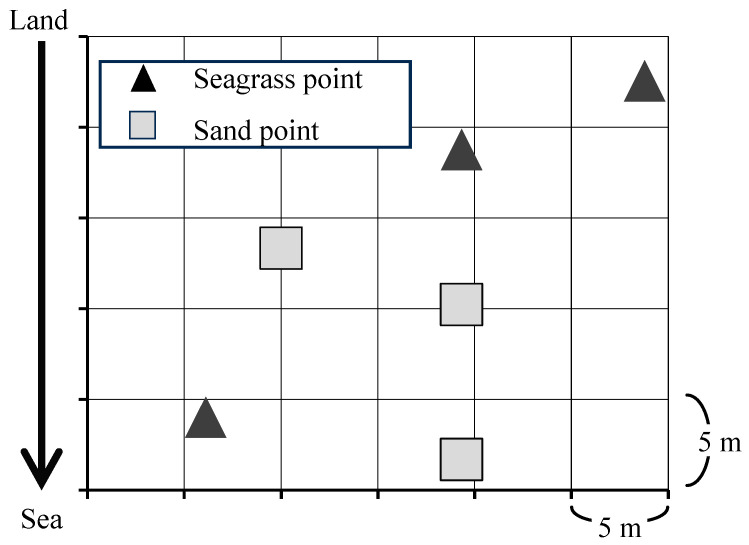
Assignment of seagrass points (S) and sand points (N).

Three *Z. japonica* patches were randomly selected from the study site, and the central point of each patch was designated as the sampling point (seagrass points). For comparison, samples were collected from nearby bare sand areas (sand points) in the same manner (Figure 3). The average values from the three points were used as representative values for the *Z. japonica* beds and sandy tidal flats. Seagrass and sandy points were separated by at least 10 m in straight lines.

POM and SOM, both of which serve as potential food sources, were collected from the sampling points in June 2006. POM was collected during high tide. To minimize disturbance, a 3 m-long sampling tube was installed during low tide. Water intake was set at each sampling site, positioned 7 cm above the sediment surface, to obtain seawater from within the seagrass bed, which corresponded to half the leaf length of *Z. japonica*. During the subsequent high tide, 3 L of seawater was collected from each site using a hand pump. From the collected water, 2 L was used to measure the suspended solid (SS) concentration. Whatman GF/C filters (Cytiva, Tokyo, Japan) (pore size 1.2 µm) were used to collect POM, which was then freeze-dried for 24 h before analyzing the SIRs and FACs.

SOM was collected during low tide. A 6 cm-diameter, 30 cm-long acrylic core sampler was used to extract the sediment vertically to minimize disturbance. After extraction, the sediment was sectioned into two layers: a surface layer (0–1 cm) and a subsurface layer (1–5 cm). Both layers were transported to the laboratory for analysis. The collected sediment was freeze-dried for 24 h and then sieved through a 1 mm mesh to separate the benthos. The samples were subsequently analyzed for ignition loss (IL), SIRs, and FAC.

The benthos surveys were conducted simultaneously. A 23 × 23 cm shovel was used to collect sediment with benthos at a depth of 30 cm. The sediment was sieved through a 1 mm mesh, and the retained material was transferred into 500 mL sample bottles along with seawater. The samples were brought back to the laboratory, temporarily frozen, and identified.

Seagrass was collected along with the sediment to a depth of 30 cm using a 23 × 23 cm shovel. The sediment containing seagrass was sieved through a 1 mm mesh, and the retained material was brought back to the laboratory. In the laboratory, the leaves and stems were immediately separated from the roots and rhizomes, and the samples were then freeze-dried and ground into a powder using a blender rinsed with methanol. The powder samples were then analyzed for SIRs and FAC.

Benthos were collected from the same site in June and August 2009, and their food sources were analyzed. The dominant benthic species were selected for analysis, and the quantity required for the analysis was collected. Target species, including *Nereididae* (polychaeta), *Batillaria cumingii* (gastropods), *Umbonium costatum* (gastropods), *Phacosoma japonicum* (bivalves), Paguroidea (hermit crabs), and *Z. japonica*, were collected. For gastropods and bivalves, the shells were removed, and only foot muscle tissue was used for analysis in the laboratory. Each species was freeze-dried for 24 h, stored in a 10 mL glass vial rinsed with methanol, and subsequently analyzed for SIRs and FAC.

### 2.2. Carbon and Nitrogen Stable Isotope Ratio Analyses

Stable C and N isotope analyses were conducted on POM collected from filters, sediments, seagrasses, and benthos, all of which were frozen at −20 °C prior to analysis. As the sediment contained a high proportion of inorganic carbonates, it was pretreated with 1 M HCL.

Analyses were performed using an isotope ratio mass spectrometer (IRMS; DELTAplus, Finnigan MAT, Bremen, Germany). The δ^13^C and δ^15^N values were calculated using the following equation:

(1) $$\delta^{13}C,\delta^{15}N=\left(R_{sample}/R_{standard}-1\right)\times 1000$$

(2) R=13C/C12,N15/N14

where *R_sample_* is the *R* of the sample, and *R_standard_* is the *R* of the standard material. Histidine (Shoko Scientific Co., Ltd., Yokohama, Japan) was used as the working standard. The analytical errors were within ±0.2‰ for both δ^13^C and δ^15^N.

### 2.3. Fatty Acid Composition Analysis

An analysis of FAC was conducted on POM collected on filters, sediments, seagrasses, and benthos, all of which were frozen at −20 °C prior to analysis. Fatty acids were extracted according to the method described by Meziane and Tsuchiya [16] and analyzed using gas chromatography (GC-17A, Shimadzu, Kyoto, Japan) with a 0.25 mm × 100 m column. Three types of standard fatty acid mixtures (i.e., PUFA No.3, Supelco™ 37 Component FAME Mix, and Bacterial Acid Methyl Esters Mix) and six long-chain fatty acid standards (SUPELCO, Bellefonte, PA, USA) were used to separate a total of 53 fatty acids. The concentration of each fatty acid was calculated based on the total lipid content, proportion of total fatty acids, and the proportion of each individual fatty acid relative to total lipid content.

### 2.4. Biomarkers of Organic Matter Sources in the Tidal Flat Ecosystem

Biomarkers of organic matter sources are compiled in Table 1, based on our previous study [17]. In the tidal flat ecosystems, the primary producers are seagrass, macroalgae, benthic microalgae (diatoms), epiphytic microalgae (diatoms and cyanobacteria), and phytoplankton (dinoflagellates and diatoms). For *Z. japonica*, rather than using the national average values, we adopted the values measured in the present study for greater accuracy. *Z. japonica* and *Z. marina* could not be distinguished based on their FAC [17]. However, because this study focused on seagrass beds dominated by *Z. japonica* and nearby bare sandy areas, *Z. japonica* was considered to have a greater influence than *Z. marina*, which is found in subtidal zones. Therefore, the seagrass fatty acid markers 18:2ω6 and 18:3ω3 were assumed to represent *Z. japonica*. Although bacterial fatty acid markers were determined, the C and N SIRs were not included. As macroalgae were rarely observed in the study area, they were excluded from the analysis. The C and N SIRs of epiphytic diatoms and cyanobacteria did not show a clear distinction from those of phytoplankton. Furthermore, their FAC could not be differentiated from those of the bacteria and benthic microalgae. Consequently, their effects were not evaluated in the present study.

### 2.5. Statistical Analysis

The data were analyzed using R v3.3.1 (R Foundation for Statistical Computing, Vienna, Austria) in RStudio v 1.0.136 (Posit, Boston, MA, USA). Normality was assessed using the Kolmogorov–Smirnov (KS) test. For comparisons between two parameters, either the t-test or Mann–Whitney U test was applied. Correlation analysis was performed using Spearman’s correlation test, and the Holm method was used for multiple comparisons. Principal component analysis (PCA) was conducted using the “vegan” package in R.

## 3. Results

### 3.1. POM and SOM

**Figure 4:**
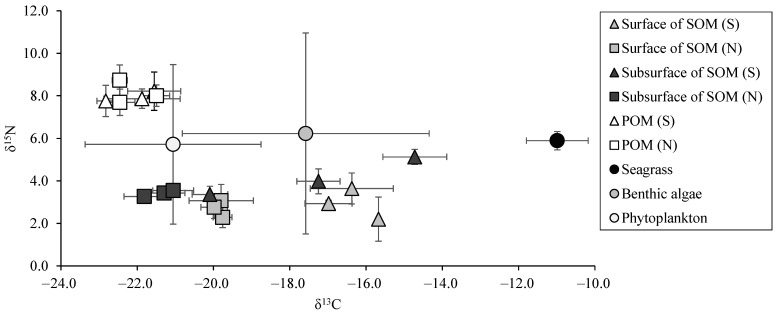
δ^13^C–δ^15^N map of particulate organic matter (POM) and sedimentary organic matter (SOM) in seagrass beds (S) and sandy areas (N). Values for the organic matter sources listed in Table 1 (i.e., seagrass, benthic algae, and phytoplankton) are also shown. Error bars represent standard deviation (SD) (*n* = 3).

The concentrations of suspended solids (SS) were compared to examine differences in POM amounts between the seagrass beds and nearby bare sandy areas. SS did not differ between the two areas (seagrass beds: 2.4 ± 0.78 mg/L, sandy areas: 2.5 ± 0.19 mg/L, *n* = 3, *p* > 0.05). Similarly, δ^13^C and δ^15^N of POM had no significant differences between the two areas (δ^13^C: seagrass beds, −22.1 ± 0.8‰; sandy areas, −22.1 ± 0.6‰; *n* = 3, *p* > 0.05, δ^15^N: seagrass beds, 8.2 ± 1.0‰; sandy areas, 8.1 ± 0.7‰; *n* = 3, *p* > 0.05) (Figure 4). The values are comparable to those of the phytoplankton (δ^13^C: −21.1 ± 2.3‰, δ^15^N: 5.7 ± 3.7‰) listed in Table 1.

The amount of SOM, measured as IL, showed no significant differences among site or sediment depths (surface in seagrass beds: 3.68 ± 0.98 mg/g, subsurface in seagrass beds: 3.12 ± 0.86 mg/g, surface in sandy areas: 2.58 ± 0.62 mg/g, subsurface in sandy areas: 2.60 ± 0.48 mg/g, *n* = 3, *p* > 0.05). However, δ^13^C and δ^15^N of SOM differed significantly (Figure 4). For both surface and subsurface layers, δ^13^C was significantly higher in the seagrass points than in the sand points (surface: seagrass beds, −16.3 ± 0.7‰; sandy areas, −19.8 ± 0.1‰; *n* = 9, *p* < 0.05; subsurface: seagrass beds, −17.4 ± 2.7‰; sandy areas, −21.4 ± 0.4‰; *n* = 9, *p* < 0.05). Additionally, δ^15^N in the subsurface layer was significantly higher than in the surface layer in both the seagrass beds and the sandy areas (*n* = 18, *p* < 0.05). Notably, δ^15^N in the subsurface layer of the seagrass beds (4.2 ± 0.9‰) was significantly higher than in the sandy areas (3.4 ± 0.1‰; *n* = 9, *p* < 0.05). Furthermore, the δ^15^N values in the seagrass beds exhibited greater variability than those in the sandy areas, possibly reflecting heterogeneity in N-related processes within the seagrass beds. This variability and its possible causes are discussed in detail in the Discussion.

**Figure 5:**
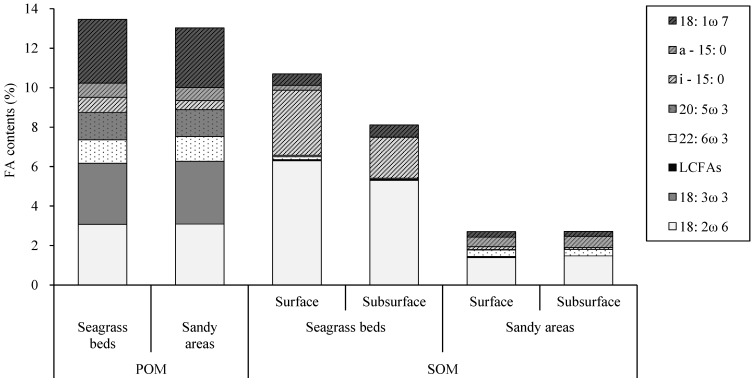
Proportions of marker fatty acids (FA) in particulate organic matter (POM) and sedimentary organic matter (SOM). FA proportions were calculated as the percentage of each marker FA relative to total FAs.

**Figure 6:**
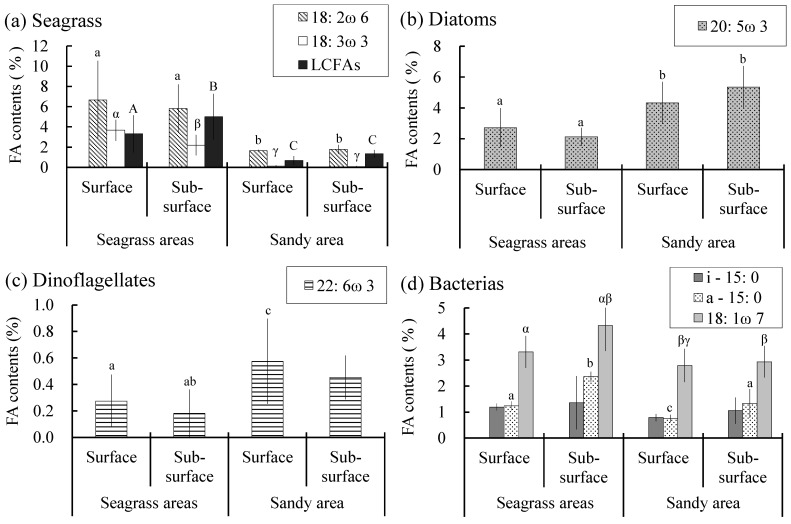
Proportions of primary producer marker fatty acids (FA) in sedimentary organic matter (SOM). FA proportions were calculated as the percentage of each marker FA relative to total FAs. Error bars represent standard deviation (*n* = 9). Different letters indicate significant differences among groups (*p* < 0.05) based on multiple comparisons. No letters are shown for comparisons in which no statistically significant differences were found.

To clarify the origins of POM and SOM, we compared the proportions of fatty acid markers (Table 1, Figure 5). The composition of POM showed little difference between the two areas, and *Z. japonica* marker fatty acids, specifically 18:2ω6 and 18:3ω3, were also detected in the POM from the sandy area. In contrast, SOM composition exhibited significant differences, prompting a more detailed examination of its origin (Figure 6). All *Z. japonica* marker fatty acids (18:2ω6, 18:3ω3, and LCFAs) were significantly more abundant in the seagrass beds than in the sandy areas (*p* < 0.05). Notably, LCFAs were particularly abundant in the subsurface layer of SOM in the seagrass beds. Diatoms were more abundant in sandy areas, whereas dinoflagellates were more prevalent in the SOM surface layer in the sandy areas. Among bacterial marker fatty acids, specifically a-15:0 and 18:1ω7 were significantly more abundant in the subsurface layer SOM of the seagrass beds (*p* < 0.05).

### 3.2. Benthic Fauna and Target Benthos

The benthic fauna collected during the 2006 survey are summarized in Table 2. The total abundance of benthic animals was significantly higher in seagrass beds (2010 ± 375 individuals/m^2^) than in the sandy areas (851 ± 312 individuals/m^2^) (*n* = 3, *p* < 0.05). If the presence of *Z. japonica* influences benthic communities, differences in abundance between seagrass beds and nearby bare sand areas are expected. Based on the differences in abundance across taxa between habitats, five target benthos were selected as indicators: (1) Nereididae (Ner), a dominant polychaete family in seagrass beds; (2) Trochidae (U. cos), a gastropod family dominant in sandy areas. All the Trochidae collected in this study were *Umbonium costatum*; (3) Batillariidae (B. cum): a gastropod family dominant in seagrass beds. All Batillariidae individuals collected in the present study were *Batillaria cumingii*; (4) Veneridae (P. jap) was the dominant bivalve family in the study area. All Veneridae collected in this study were *Phacosoma japonicum*; and (5) Paguroidea (Pag), hermit crabs were more abundant in seagrass beds.

**Figure 7:**
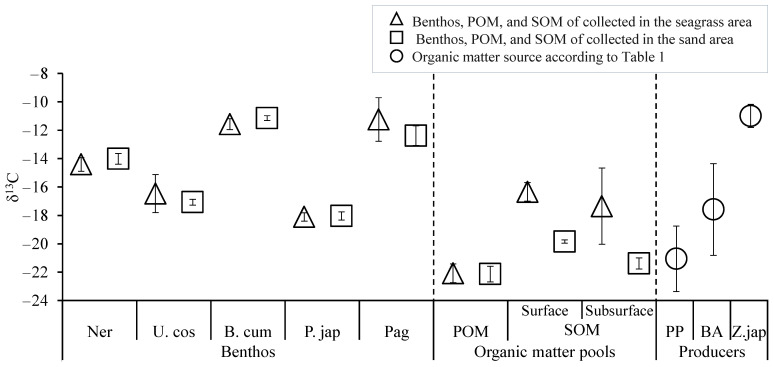
δ^13^C values of target benthos—Nereididae (Ner), Trochidae (U.cos), Batillariidae (B.cum), Veneridae (P.jap), and Paguroidea (Pag)—as well as organic matter pools (i.e., POM, surface SOM and subsurface SOM, and organic matter sources (i.e., phytoplankton [PP], benthic algae [BA], and seagrass [*Z. japonica*: Z.jap]) based on data in Table 1. Error bars represent standard deviation (SD).

Organic matter sources of the target benthos were estimated using stable C isotope ratios. The δ^13^C values of the target benthos, along with those of the three assumed organic matter sources (i.e., phytoplankton, benthic algae, and seagrass), are presented in Figure 7. The δ^13^C values of the target benthos exhibited minimal difference between individuals collected from the seagrass beds and sandy areas. Their δ^13^C values were consistently higher than those of phytoplankton but lower than those of *Z. japonica*. Among the target benthos, *B. cumingii* and Paguroidea exhibited δ^13^C values similar to those of *Z. japonica*, suggesting that they consume seagrass-derived organic matter.

**Figure 8:**
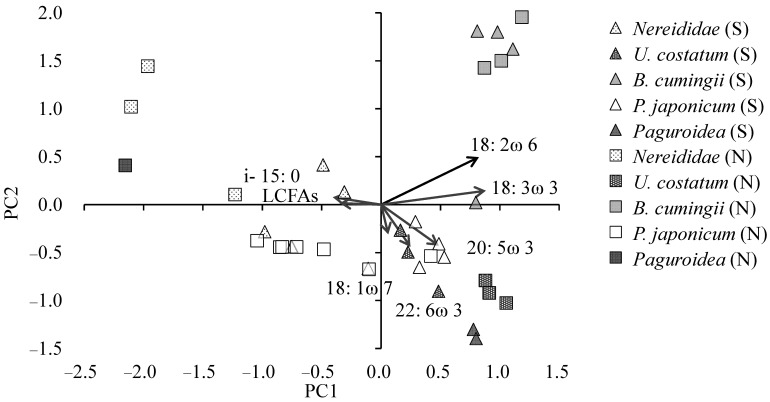
Results of principal component analysis (PCA) based on the fatty acid composition of the target benthos collected in seagrass beds (S) and sandy areas (N).

To further assess the organic matter sources in the target benthos, we analyzed the fatty acid markers of primary producers and bacteria in the target benthos. The FACs of the target benthos are shown in Table 3. We then conducted PCA on the fatty acid markers of the target benthos collected from seagrass beds and nearby bare sandy areas (Figure 8). The cumulative contributions of PC1 and PC2 were 0.40. Unlike the stable isotope ratio results, the FAC revealed clear differences between individuals collected from seagrass beds and sandy areas. Although there was some variability, Nereididae collected from both habitats were mainly located in the negative regions of PC1 and PC2, showing strong associations with i-15:0 and LCFAs. Those collected from seagrass beds tended to exhibit values closer to *Z. japonica*. Their PC1 scores were relatively higher than those from sandy areas, suggesting stronger associations with seagrass marker fatty acids such as 18:2n6 and 18:3n3.

*U. costatum* was strongly associated with 20:5ω3 and 22:6ω3. *U. costatum* specimens collected from seagrass beds tended to be positioned slightly closer to i-15:0 and LCFAs than those from sandy areas, suggesting a minor influence of seagrass-derived organic matter. *B. cumingii* exhibited highly characteristic values, showing little difference between seagrass beds and sandy areas, with strong associations with 18:2ω6 and 18:3ω3. *P. japonicum* was positioned near 22:6ω3, although there was considerable variation among individuals. Additionally, Paguroidea showed notable differences between seagrass beds and sandy areas. Those collected in the seagrass beds were strongly associated with 20:5ω3 and 22:6ω3, whereas those collected in the sandy areas exhibited stronger relationships with i-15:0 and LCFAs.

## 4. Discussion

Among the target benthos examined in the present study, *B. cumingii*, an epifaunal gastropod, showed high assimilation of organic matter derived from the intertidal seagrass *Z. japonica*, as indicated by its C SIR (Figure 7) and FAC (Figure 8). In contrast, the consumption and assimilation of diatoms and dinoflagellates appeared to be lower than that of other target benthos, suggesting a higher dietary dependence on *Z. japonica* than on other target benthos. Kharlamenko et al. [13] demonstrated that Batillaria in subtidal *Z. marina* beds rely on seagrass-derived organic matter as an important food source, which is consistent with our findings. Additionally, previous studies have reported that epipelic diatoms attached to seagrass blade surfaces are frequently observed in the gut contents of Batillaria [25]. Several studies from the 1980s have indicated that Batillaria feed on epipelic diatoms in surface sediments [26,27]. However, Koike et al. [25] pointed out that their gut content analysis method involved the removal of organic matter using 17% H_2_O_2_, which prevented direct observation of seagrass and detritus. Furthermore, our study revealed that *B. cumingii* collected not only from seagrass beds but also from bare sandy areas exhibited a notably high abundance of seagrass marker fatty acids, specifically 18:2ω6 and 18:3ω3, compared to other benthos (Figure 8). This suggests that *B. cumingii* in tidal flats have a high dietary requirement for these fatty acids.

Conversely, although Paguroidea (hermit crabs) in tidal flats exhibited similarly high δ^13^C values as *B. cumingii* (Figure 7), the results of FAC analysis (Figure 8) did not indicate a high dietary dependence on seagrass-derived organic matter. Hermit crabs are opportunistic feeders; however, their specific dietary sources remain unclear [28]. Despite efforts to collect more individuals, only a small number of individuals were analyzed, and species-level identification was not conducted in the present study, highlighting the need for further investigations into species-specific dietary preferences.

The δ^13^C values of Nereididae were higher than those of SOM (Figure 7) and were closer to those of the intertidal seagrass *Z. japonica.* However, among the three *Z. japonica* marker fatty acids, the relationship with 18:2ω6 and 18:3ω3 was weak, while a remarkably strong association was observed with LCFAs. Additionally, Nereididae exhibited a strong relationship with bacterial marker fatty acids, specifically i-15:0 and 18:1ω7. Focusing on SOM, the total abundance of all seagrass marker fatty acids (18:2ω6, 18:3ω3, and LCFAs) showed a significant positive correlation with the total abundance of bacterial marker fatty acids (*n* = 36, *r* = 0.51, *p* < 0.01). In contrast, no correlation was observed between seagrass marker fatty acids and diatom or dinoflagellate marker fatty acids (*n* = 36, *p* > 0.05). In the subtidal zone, *Z. marina* forms a rhizosphere that provides a habitat for numerous bacteria [29,30]. This suggests that *Z. japonica* in the intertidal zone also developed a rhizosphere in its subsurface sediments, providing a habitat for bacteria. This is also supported by δ^15^N values. Although δ^15^N values within seagrass beds exhibited some variation, they tended to be higher than in nearby bare sandy areas. This increase may reflect microbial processes such as denitrification occurring in subsurface sediments, potentially stimulated by the presence of *Z. japonica* detritus, which serves as an organic substrate. One possible factor contributing to the increase in stable N isotope ratios is denitrification [31], which suggests that denitrification using *Z. japonica* detritus as a substrate in subsurface sediments may occur within seagrass beds. Furthermore, the high δ^13^C values in Nereididae and the abundance of bacterial marker fatty acids strongly suggest that Nereididae primarily feed on *Z. japonica* detritus, likely via bacteria that utilize seagrass-derived organic matter in subsurface sediments. Previous studies on seagrass beds in subtidal zones have examined the feeding habits of Nereididae species such as *Nereis diversicolor*, suggesting that they are carnivorous [21]. However, species within the family Nereididae exhibit a wide range of feeding strategies, including carnivory, herbivory, and deposit feeding, even within the same species [32,33]. In the present study, the proportion of polychaetes in the total benthic fauna was higher in intertidal seagrass beds than in nearby bare sandy areas (Table 2). Previous studies have also reported the enrichment of polychaetes in intertidal seagrass beds [17], which was also observed in the present study (Table 2). The findings are consistent with those obtained for Nereididae feeding on *Z. japonica* detritus.

*U. costatum* and *P. japonicum* exhibited low dietary dependence on intertidal seagrass *Z. japonica*-derived organic matter. Both species are filter feeders and primarily consume POM or resuspended surface SOM [34,35]. Although *Z. japonica*-derived organic matter was present in the POM and SOM (Figure 5), its contribution as a food source for *U. costatum* and *P. japonicum* was minimal. Miyaji et al. [36] demonstrated a positive correlation between phytoplankton abundance in the seawater column and growth rates of *P. japonicum*. This suggests that filter-feeding benthos such as *U. costatum* and *P. japonium* have low dietary requirements for intertidal seagrass-derived organic matter.

Although the influence of *Z. marina*, which grows in the subtidal zone of the study site, cannot be completely ruled out, this study demonstrated that seagrasses, represented by *Z. japonica*, which are highly productive in tidal flat zones, play an important role in intertidal ecosystems. However, an experimental approach such as a mesocosm study would be useful for determination of the precise trophic importance of *Z. japonica*.

The results of the present study show that, similar to subtidal seagrass ecosystems, some benthos inhabiting tidal flats utilize either seagrass-derived organic matter or bacteria nourished by intertidal seagrass as a food source. In contrast, some benthos showed low dietary dependence on seagrass organic matter. The findings suggest that the conservation and restoration of intertidal seagrass beds, rather than only subtidal beds, could directly contribute to the enhancement of biodiversity in tidal flat ecosystems. Hadded et al. [37] demonstrated that plant diversity promotes ecosystem stability by increasing invertebrate diversity and abundance in aquatic ecosystems. Thus, our findings suggest that, in tidal flats, the conservation and restoration of intertidal seagrass beds, in addition to traditional sandy habitats, may play a crucial role in maintaining the biological stability of these ecosystems.

## 5. Conclusions

This study aimed to clarify the influence of intertidal seagrass on benthos inhabiting tidal flats from a food resource supply perspective. The findings will contribute to the conservation and restoration of resilient intertidal flat ecosystems that are highly affected by human activity. The key findings of this study are as follows:

(1) *Batillaria cumingii*, an epifaunal mud snail inhabiting intertidal flats, actively consumes organic matter derived from intertidal seagrass *Z. japonica*. *Z. japonica*-derived organic matter was present not only in the surface sediments of seagrass beds, but also in sandy areas. Therefore, the presence of nearby intertidal seagrass beds provides a favorable habitat within the intertidal flats of *B. cumingii*.
(2) Nereididae, which inhabit intertidal flats, have been suggested to primarily consume bacteria and seagrass detritus. A significant correlation was observed between *Z. japonica* and bacteria, with bacterial abundance being higher in the intertidal seagrass beds. This indicates that intertidal seagrass beds serve as suitable habitats for Nereididae within intertidal flats.
(3) Although *Z. japonica*-derived organic matter was present in the POM of both the intertidal seagrass beds and nearby bare sandy areas, *Umbonium costatum* and *Phacosoma japonicum*, filter feeders inhabiting intertidal flats, showed little utilization.

These findings demonstrate that, similar to subtidal seagrass ecosystems, some benthos in tidal flats use intertidal seagrass-derived organic matter and bacteria nourished by seagrass as food sources. In contrast, some benthos exhibit a low dietary dependence on seagrass-derived organic matter. The findings suggest that the conservation and restoration of intertidal seagrass beds, rather than exclusively subtidal beds, could directly contribute to the enhancement of biodiversity in tidal flat ecosystems.

## Figures and Tables

**Table 1:** Organic matter sources, their markers (δ^13^C and δ^15^N values and fatty acids), and references used in this study. LCFA indicates long-chain fatty acids. SD indicates standard deviation.

Organic Matter Source	Producer	δ^13^C (SD)	δ^15^N (SD)	Ref.	Fatty Acid	Ref.
Seagrass	*Z. japonica*	−11.0 (0.8)	5.9 (0.4)	This research	18: 2ω6 18: 3ω3 LCFAs	[17]
Benthic algae	Diatoms	−17.6 (3.2)	6.2 (4.7)	[18,19,20]	20: 5ω3	[10,13,21]
Phytoplankton	Diatoms and Dinoflagellates	−21.1 (2.3)	5.7 (3.7)	[13,20,22,23]	Diatom: 20: 5ω3	[10,13,21]
					Dinoflagellates: 22: 6ω3	[10,13,24]
Soil bacteria					i-15:0 a-15:0 18: 1ω7	[10,13,16,21]

**Table 2:** Density of benthic fauna collected during the 2006 survey (individuals/m^2^).

	Seagrass Points		Sand Points			Seagrass Points		Sand Points	
	mean	SD	mean	SD		mean	SD	mean	SD
Polychaeta					Gastropoda				
Nereididae	485	(743)			Trochidae	76	(68)	466	(317)
Phyllodocidae	13	(22)			Batillariidae	32	(55)		
Nephtyiclae			25	(11)	Naticidae	6	(11)		
Glyceridae			6	(11)	Nassariidae	139	(95)	13	(22)
Lumbrineridae	32	(22)	13	(11)	Cylichnidae			6	(11)
Onuphidae	6	(11)	19	(33)	Bivalvia				
Spionidae			63	(58)	Tellinidae	25	(44)	19	(19)
Cirratulidae	25	(11)			Veneridae	271	(39)	170	(168)
Capitellidae	50	(39)	32	(55)	Myidae	44	(76)		
Maldanidae	6	(11)			Malacostraca				
Terebellidae	32	(55)	6	(11)	Gammaridae	50	(39)	13	(11)
					Cirolanidae	25	(44)		
					Paguroidea	687	(398)		
					Barchyuran	6	(11)		

**Table 3:** Fatty acid composition (FAC, %) of target benthos—Nereididae (Ner), Trochidae (U.cos), Batillariidae (B.cum), Veneridae (P.jap), and Paguroidea (Pag)—collected from seagrass areas and sandy areas.

	Seagrass Area										Sandy Area									
	Ner		U.cos		B.cum		P.jap		Pag		Ner		B.cum		U.cos		P.jap		N_Pag	
	*n* = 3		*n* = 4		*n* = 3		*n* = 6		*n* = 2		*n* = 3		*n* = 3		*n* = 3		*n* = 7		*n* = 1	
	mean	SD	mean	SD	mean	SD	mean	SD	mean	SD	mean	SD	mean	SD	mean	SD	mean	SD	mean	SD
18:2n6c	-	-	1.7	(0.3)	3.6	(0.4)	0.8	(0.6)	2.1	(0.0)	-	-	4.3	(0.9)	1.8	(0.5)	0.5	(0.6)	-	-
18:3n3	-	-	1.4	(0.4)	1.6	(0.3)	0.6	(0.7)	1.3	(0.0)	-	-	2.1	(0.2)	1.8	(0.1)	0.4	(0.6)	-	-
LCFAs	3.2	(5.6)	0.8	(1.4)	0.3	(0.6)	-	-	-	-	2.5	(1.6)	-	-	-	-	-	-	-	-
22:6n3	0.9	(0.9)	5.8	(0.5)	4.9	(1.5)	17.6	(2.8)	6.8	(0.7)	0.4	(0.7)	3.5	(0.3)	5.6	(0.3)	11.0	(5.6)	-	-
20:5n3	17.8	(3.8)	14.0	(0.4)	11.0	(0.1)	13.7	(1.3)	15.7	(0.4)	9.2	(7.1)	10.3	(1.0)	16.2	(0.2)	9.8	(4.1)	-	-
i-15:0	-	-	-	-	-	-	-	-	-	-	-	-	-	-	-	-	-	-	1.3	-
a-15:0	1.6	(0.1)	-	-	2.7	(1.2)	-	-	1.3	(0.1)	3.7	(1.6)	1.3	(0.1)	-	-	-	-	2.5	-
18:1n7	11.9	(1.9)	8.5	(0.9)	4.7	(1.5)	3.4	(0.6)	6.8	(0.3)	6.0	(3.5)	3.9	(0.7)	9.3	(0.3)	4.6	(0.6)	1.2	-

## Data Availability

The data for this research are available from the corresponding author upon request.
